# Impact of sex, MHC, and age of recipients on the therapeutic effect of transferred leukocytes from cancer-resistant SR/CR mice

**DOI:** 10.1186/1471-2407-9-328

**Published:** 2009-09-15

**Authors:** John R Stehle, Michael J Blanks, Gregory Riedlinger, Jung W Kim-Shapiro, Anne M Sanders, Jonathan M Adams, Mark C Willingham, Zheng Cui

**Affiliations:** 1 Department of Pathology, Wake Forest University School of Medicine Winston-Salem, North Carolina 27157, USA; 2 Molecular Genetics & Genomics Program, Wake Forest University School of Medicine Winston-Salem, North Carolina 27157, USA; 3 Department of Cancer Biology, Wake Forest University School of Medicine Winston-Salem, North Carolina 27157, USA

## Abstract

**Background:**

Spontaneous Regression/Complete Resistant (SR/CR) mice are resistant to cancer through a mechanism that is mediated entirely by leukocytes of innate immunity. Transfer of leukocytes from SR/CR mice can confer cancer resistance in wild-type (WT) recipients in both preventative and therapeutic settings. In the current studies, we investigated factors that may impact the efficacy and functionality of SR/CR donor leukocytes in recipients.

**Results:**

In sex-mismatched transfers, functionality of female donor leukocytes was not affected in male recipients. In contrast, male donor leukocytes were greatly affected in the female recipients. In MHC-mismatches, recipients of different MHC backgrounds, or mice of different strains, showed a greater negative impact on donor leukocytes than sex-mismatches. The negative effects of sex-mismatch and MHC-mismatch on donor leukocytes were additive. Old donor leukocytes performed worse than young donor leukocytes in all settings including in young recipients. Young recipients were not able to revive the declining function of old donor leukocytes. However, the function of young donor leukocytes declined gradually in old recipients, suggesting that an aged environment may contain factors that are deleterious to cellular functions. The irradiation of donor leukocytes prior to transfers had a profound suppressive effect on donor leukocyte functions, possibly as a result of impaired transcription. The cryopreserving of donor leukocytes in liquid nitrogen had no apparent effect on donor leukocyte functions, except for a small loss of cell number after revival from freezing.

**Conclusion:**

Despite the functional suppression of donor leukocytes in sex- and MHC-mismatched recipients, as well as old recipients, there was a therapeutic time period during the initial few weeks during which donor leukocytes were functional before their eventual rejection or functional decline. The eventual rejection of donor leukocytes will likely prevent donor leukocyte engraftment which would help minimize the risk of transfusion-associated graft-versus-host disease. Therefore, using leukocytes from healthy donors with high anti-cancer activity may be a feasible therapeutic concept for treating malignant diseases.

## Background

Spontaneous regression/complete resistant (SR/CR) mice are a line of cancer-resistant mice that are capable of resisting large doses of transplanted lethal cancer cells [1-3]. The basis for this powerful resistance to cancer cells is leukocytes that are capable of detecting, infiltrating, and killing cancer cells within a few hours of exposure [1]. The major component of this anti-cancer response resides in granulocyte, monocyte, and natural killer cell fractions that constitute innate cellular immunity [1,2]. The adoptive transfer of donor leukocytes from SR/CR mice can confer protection against future exposures to cancer cells, as well as the elimination of established malignancies without any further manipulation in cancer-sensitive wild-type (WT) recipient mice [2].

These prior studies suggest that it may be possible to develop a similar leukocyte transfer platform for humans if we can identify humans with anti-cancer activity similar to the SR/CR mice to serve as leukocyte donors. However, the prior adoptive transfers of donor leukocytes in these mice were considerably different from what would be necessary in human cancer patients. The leukocyte transfers in mice were done in MHC-matched and relatively young recipients [2]. In the potential human setting, the circumstance could be vastly different.

First, it is not known how much of an effect a sex mismatch would have on the donor leukocytes. It is possible for a donor leukocyte treatment to involve donors that are a different sex than the recipient. While female donor leukocytes in a male recipient are not expected to cause substantial problems, it is reasonable to expect some issues with male donor leukocytes in female recipients because of the existence of unique proteins associated with y-chromosome gene expression [4]. However, it is unclear how much of an impact this incompatibility would have on the functionality of male donor leukocytes, and if this mismatch is enough to render the donor leukocytes ineffective in the recipients.

Second, in the human setting, a long-term engraftment of donor leukocytes, especially donor T lymphocytes, should be avoided in order to minimize the possibility of transfusion-associated graft-versus-host diseases (TA-GVHD) [5]. The long-term engraftment of donor leukocytes in immunocompetent individuals is usually caused by an incomplete but near match of human lymphocyte antigens (HLA) between the donor leukocytes and the recipients, such as donor leukocytes from blood relatives [6,7]. One way to help avoid long-term engraftment is by transferring donor leukocytes into immunocompetent recipients that have been selected based on a complete HLA mismatch. The complete HLA-mismatch between donors and recipients should cause the donor leukocytes to be rejected in several weeks to several months. Our prior mouse transfer experiments have shown that the donor leukocytes can work in a rapid manner over the course of several days, or 2-3 weeks in the MHC-matched background. In the setting of a human leukocyte transfer, a complete HLA-mismatched scenario would minimize the risk for TA-GVHD in an immunocompetent individual due to the timely rejection of the donor cells before the occurrence of TA-GVHD. However, it is unknown whether there is an initial therapeutic window before the donor leukocytes are eventually rejected due to the HLA-mismatch, in which the donor leukocytes may still offer an effective time period in which the donor leukocytes are functional.

Third, the transfers in the prior studies were done primarily with young mice as the recipients and donors [2]. The scenario in the human setting would most likely be much more complicated. Since there is extensive immune dysfunction that occurs as one ages [8,9], it is assumed that younger individuals would have better immune cell function and, therefore, make better donors. Even if younger individuals were used as donors, the leukocytes would be transferred into cancer patients who consist mainly of an older population [10]. Based on prior results, it is thought that the aged cellular environment may contain circulating factors that are inhibitory to the functions of younger cells [11]. However, it is currently unknown how much of an impact the older recipient environment would have on the functionality of younger donor leukocytes.

In this paper, we describe the results of studies that determine the impact of sex, MHC, and age on the therapeutic effects of SR/CR donor leukocytes in a lethal cancer model.

## Results and discussion

### Transferred anti-cancer activity in MHC-and sex-mismatched recipients

**Figure 1:**
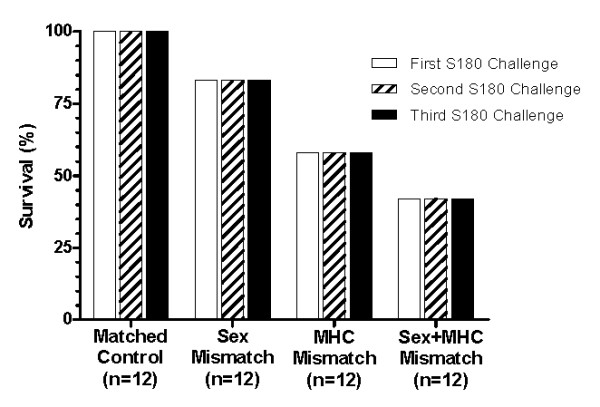
**Functionality of donor leukocytes in mismatched recipients**. The pooled donor leukocytes were evenly given to the recipients intraperitoneally (IP). After transfers of donor leukocytes, recipient mice were challenged with 1 × 10^6 ^S180 cells IP. The percent survival of the recipient mice was presented to indicate the functionality of the transferred donor leukocytes after the first challenge (left bars, 1 day after the transfer), second challenge (middle bars, 6 weeks after the transfer) and third challenge (right bars, 12 weeks after the transfer). All recipient mice were WT mice that were sensitive to S180 challenges. For the matched controls, 6 male BALB/c recipients were given male BALB/c SR/CR donor leukocytes and 6 female BALB/c recipients were given female BALB/c SR/CR donor leukocytes. For the sex mismatch, 6 female BALB/c recipients were given male BALB/c SR/CR donor leukocytes; 6 male BALB/c recipients were given female BALB/c SR/CR donor leukocytes. For the MHC mismatch, 6 male C57BL/6 mice were given male BALB/c SR/CR leukocytes and 6 female C57BL/6 mice were given female BALB/c SR/CR leukocytes. For the sex and MHC double mismatch, 6 female C57BL/6 mice were given male BALB/c SR/CR leukocytes and 6 male C57BL/6 mice were given female BALB/c SR/CR leukocytes. The percent survival for each of the adoptive transfers remains unchanged after each subsequent S180 challenge. The overall survival for each experimental group was as follows: matched is 100%, sex mismatch is 83%, MHC mismatch is 58%, and MHC + sex mismatch is 42%.

**Figure 2:**
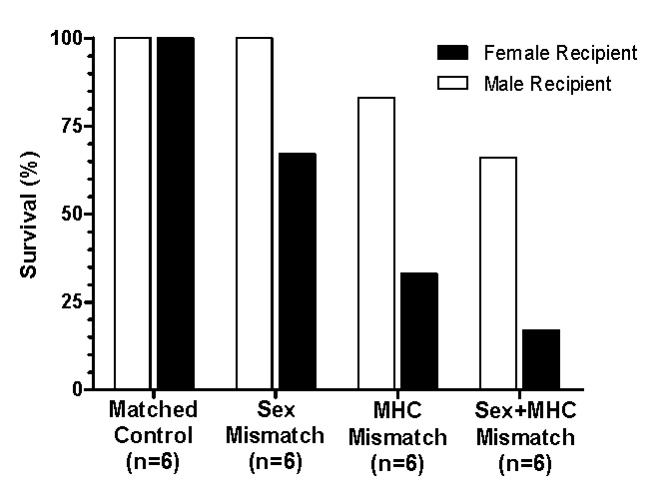
**Functionality of donor leukocytes in recipients of different sexes**. These are the same experimental conditions as those described in Figure 1. The left bars of each column are survival percentages of male recipient mice and right bars are those of female recipient mice after the completion of all three S180 IP challenges. The female recipients display a lower survival in all mismatched groups. The percent survival for each group is as follows: male 100% versus female 67% for the sex-mismatch, male 83% versus female 33% for MHC-mismatch, and male 67% versus female 17% for sex- and MHC-mismatch.

To determine the impact of different recipient MHC and sex backgrounds on transferred anti-cancer activity, we performed a set of experiments where the mice were intentionally mismatched for strain and sex. For the sex-mismatch, SR/CR BALB/c male donor leukocytes were transferred into WT BALB/c female recipients, or SR/CR BALB/c female donor leukocytes were transferred into WT BALB/c males. For the MHC-mismatch, SR/CR BALB/c female donor leukocytes were transferred into WT C57BL/6 female recipients, or SR/CR BALB/c male donor leukocytes were transferred into WT C57BL/6 male recipients. For the sex- and MHC- double mismatch, SR/CR BALB/c male donor leukocytes were transferred into female WT C57BL/6 recipients, or SR/CR BALB/c female donor leukocytes were transferred into WT C57BL/6 males. All WT recipient mice were then challenged with 1 × 10^6 ^S180 cells, 24 hours after the adoptive transfer, to evaluate the anticancer activity of the transferred leukocytes. The surviving mice were then challenged with 1 × 10^6 ^S180 two more times with 5-6 week intervals between the injections. As expected, the sex- and MHC-matched controls showed 100% overall survival after all three S180 challenges (Figure 1). The sex-mismatch mice resulted in an overall survival of 83% (Figure 1) after three S180 challenges. The MHC-mismatch resulted in a reduction of overall survival (58%) which was maintained for each of the subsequent tumor challenges (Figure 1). When the recipients were mismatched for both MHC and sex, the overall survival dropped to approximately 42%, but remained unchanged after the first tumor challenge. Interestingly, when the recipients were evaluated by sex (Figure 2), the female recipients displayed a lower survival in all mismatched groups tested. When the transfers were mismatched for sex, 100% survival was observed in the male recipients with only 67% survival in the female recipients (Figure 2). When the recipients were MHC-mismatched, the male recipients had 83% survival while the females only had 33% survival (Figure 2). Finally, in the double-mismatch for sex and MHC, male recipients had a higher survival percentage (67%) when compared to the female recipients (17%) (Figure 2). All of the failed adoptive transfers were observed during the first challenge with S180 since the percent survival remained unchanged throughout the second and third challenges for all groups evaluated (Figure 1).

**Figure 3:**
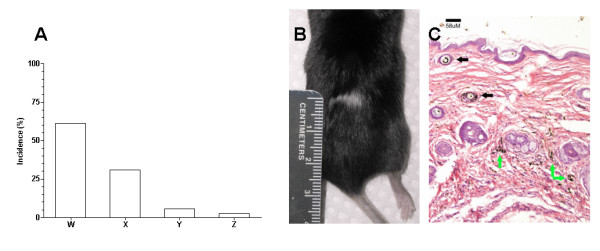
**Side effects associated with the adoptive transfers**. **A**) Incidental rates as a result of the leukocyte transfer. "W" (61%) refers to successful transfer of anticancer activity and protection of the recipient mice from subsequent challenges with S180. "X" (31%) refers to the animals that developed S180-induced ascites presumably due to the rejection of donor leukocytes by the recipients. "Y" (5.5%) refers to the deaths of recipient mice after the transfer of donor leukocytes due to unknown reasons, but not due to development of ascites. "Z" (2.5%) refers to an incident of fur discoloration post-adoptive transfer potentially representing graft-versus-host disease. **B**) An image of fur discoloration as a result of donor leukocyte transfers. **C**) Skin histology under discolored fur indicates post inflammatory pigment incontinence. The black arrows point to areas with normal appearing pigmentation while the green arrows point to the areas of pigment incontinence in which the pigment localized in macrophages at the periphery of the follicles.

Out of the 36 mice that received mismatched adoptive transfers, 61% of the mice remained healthy with no signs of malignancy or GVHD development (Figure 3A, Column W). Following the first challenge with S180 cells, 31% of the recipients developed ascites (Figure 3A, Column X) which suggests that the donor leukocytes were rejected in the recipient. Approximately 5.5% of the mismatched mice died of unknown causes while approximately 2.5% of the mismatched mice (n = 1) displayed fur discoloration which is suggestive for GVHD (Figure 3A, Column Z, Figure 3B, C). This one mouse had a discoloration of fur (Figure 3B) that appeared approximately 14 days post-adoptive transfers. Upon histological evaluation, post inflammatory pigment incontinence was observed (Figure 3C). The black arrows point to areas with normal appearing pigmentation while the green arrows point to areas of pigment incontinence, in which the pigment was localized in macrophages at the periphery of the follicles. This observation suggests a prior inflammatory response that resolved prior to the end of the study. In the human setting, transfusion associated GVHD is a rare complication that is associated with fever, rash, abdominal pain, vomiting and diarrhea and is usually fatal [5,6]. These symptoms were not found in the treated mice; however the fur discoloration is suggestive of a GVHD reaction.

### Transferred anti-cancer activity in recipients of different ages

**Figure 4:**
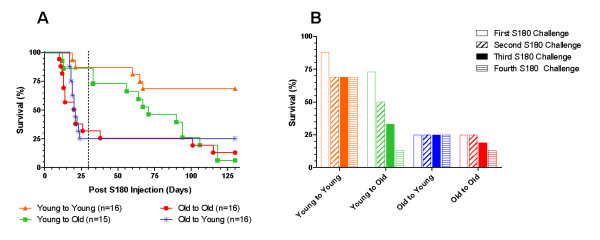
**Impact of leukocyte age and recipient age on donor leukocyte functionality**. **A**) Survival curves. The ages of "young" C57BL/6 SR/CR donors were 4.8 ± 0.5 months and "old" donors were 22.9 ± 3.5 months. The ages of "young" C57BL/6 WT recipients were 2 months and "old" recipient mice were 24 months. Survival percentages of recipient mice are shown: young to young (Orange triangle, n = 16), young to old (Green square, n = 15), old to old (Red circle, n = 16) and old to young (Blue asterisk, n = 16). The adoptive transfer recipients were then challenged 1 day after the adoptive transfer, then approximately every 30 days thereafter for the survivors. The area to the left of the dashed line represents the impact that the age of the donor leukocytes has on the survival of the recipient. **B**) Bar chart. The same experimental conditions as described in figure 4A after each S180 challenge. The survival for each experimental group after the subsequent challenges was as follows: First S180 challenge: 88% for young to young, 73% for young to old, 25% for old to young, and 25% for old to old; Second S180 challenge: 69% for young to young, 50% for young to old, 25% for old to young, and 25% for old to old; Third S180 challenge: 69% for young to young, 33% for young to old, 25% for old to young, and 13% for old to old; Fourth S180 challenge: 69% for young to young, 13% for young to old, 25% for old to young, and 13% for old to old.

Since most cancers occur in an aged population [10], we wanted to evaluate what impact this could have on adoptively transferred leukocytes. Leukocytes were transferred from young (4.75 ± 0.5 months) or old (22.88 ± 3.54 months) SR/CR C57BL/6 donors to young (3 months) or old (24 months) C57BL/6 WT mice, with sex and MHC are both matched. The recipient mice were then initially challenged with 1 × 10^6 ^S180 24 hours after the adoptive transfer, followed by three subsequent S180 injections with 5-6 week intervals. As seen in the survival curve (Figure 4A), the control group (young donor leukocytes to young recipients) resulted in the highest overall survival percentage among the four groups. When an aged mouse was used as either the donor or the recipient, the survival was drastically reduced in all three groups, either immediately or over time following the adoptive transfer (Figure 4A). When the survival was evaluated after each S180 injection, three of the four groups (young donor to young recipient, old donor to old recipient, and old donor to young recipient) showed minimal changes after the initial S180 challenge (Figure 4B). In contrast to the other three groups, when young cells were transferred to an old environment, the recipients initially displayed a high percent survival, but then showed a continual decline after each subsequent challenge with S180 presumably due to the impact of the aged host environment on the donor leukocytes (Figure 4B).

**Figure 5:**
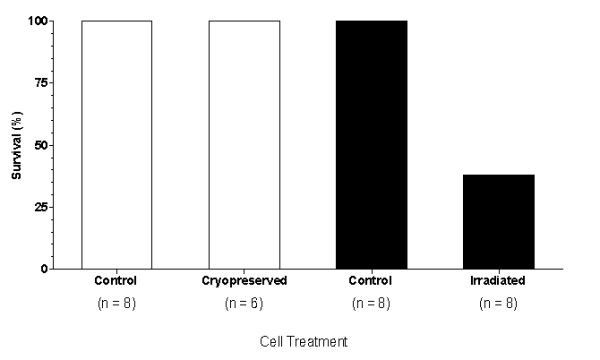
**Effects of cryopreservation and irradiation on the donor leukocytes before transfers**. The procedures of cryopreserving and irradiating donor leukocytes were described in the section of "materials and methods". After each adoptive transfer the groups were challenged with 1 × 10^6 ^S180. The percent survival after the S180 challenges is shown. The untreated controls for both groups are 100% survival. The cryopreserved leukocytes also result in 100% survival while the leukocytes irradiated at 25 Gray results in 38% survival.

### Effect of irradiation on the function of transferred leukocytes

The irradiation of cells can be used to help minimize the risk of GVHD in adoptive immunotherapy settings [12]. The irradiation irreversibly damages the DNA of the leukocytes, preventing their cell division without killing the cells. However, it is unclear if this process may have an immediate affect on the anticancer killing activity of the leukocytes. To evaluate this in our model, leukocytes of the spleen were harvested from C57BL/6 SR/CR mice, pooled, and then split into two groups. One group was irradiated (25Gy), while the other served as the nonirradiated control group. Each group was transferred to C57BL/6 WT mice and challenged 24 hours later with 1 × 10^6^S180. The nonirradiated controls resulted in 100% survival, while the irradiated group displayed a reduction to only 38% survival (Figure 5). Overall, irradiation negatively impacted, but did not abolish the cancer killing activity of the SR/CR leukocytes.

### Effect of cryopreservation on the function of donor leukocytes

Cryopreservation is a common practice for storing cells [13,14]. Most of the time the viability of stored cells is minimally affected by freezing and thawing, it is not clear whether a specific functionality, such as the anticancer activity of SR/CR donor leukocytes, can be affected by cryopreservation. Leukocytes were harvested from SR/CR donor mice, pooled and divided into two groups. One was given directly to WT mice as a non-frozen control, while the other cells were frozen for approximately 30 days. Then, these cells were thawed and given to another group of WT mice. There were 6 mice used for the post-cryopreservation due to the loss of a tube of cells during the retrieval process. By using 6 for the group, then the number of cells given per recipient was closer to the control recipients' numbers (within 2 × 10^6 ^leukocytes). Both groups of WT mice were challenged with 1 × 10^6 ^S180 cells 24 hours after the designated adoptive transfers. Even with the slightly reduced number of leukocytes delivered after cryopreservation, both groups showed identical survival (100%) after a challenge with S180 (Figure 5). Therefore cryopreservation appeared to have no negative impact on the cancer killing activity of the SR/CR leukocytes.

## Conclusion

It has long been theorized that clinically significant cancer is caused by a defective immune system which results in a loss of activity capable of removing cancer cells that are continuously being generated [15]. However, adoptive transfer of innate leukocytes from donors with a validated high level of cancer-killing activity to cancer patients for therapeutic purposes is a new concept. In comparison, the major difference is that most conventional cancer immunotherapies try to stimulate a possibly damaged immune system of cancer patients, whereas this new concept attempts to supplement the damaged components of the immune system with ones with enhanced activity. Prior studies in SR/CR mice suggested that such a cancer surveillance system can be mediated by leukocytes of the innate immune system [1,2]. Their powerful immune system is capable of eradicating a large number of malignant cells that otherwise would be lethal. It may be possible to develop similar therapeutic concepts in the human setting. The studies described in this paper investigated the factors that may have substantial impact on the efficacy of donor leukocytes therapeutically.

Our studies indicated that male recipients had no apparent inhibitory effect on the female donor leukocyte functionality. On the other hand, transfers of male donor leukocytes provoked a leukocyte rejection mechanism in female recipients to a considerable degree. This observation is consistent with the concept that y-chromosome-associated proteins correlate with chronic graft-versus host disease [4] and, therefore, may provoke a time-dependent transplant rejection in female recipients. However, even with the loss of donor leukocyte function due to the rejection mechanism, there may still be an initial therapeutic window during the first few days or even weeks before the donor leukocytes are rejected. Therefore, the transfer may still be effective in both sex-mismatched settings at least during the first few weeks.

It is also clear that an MHC-mismatch can have a substantial impact on the transferred leukocytes. This finding is not surprising since it is well known that different MHC molecules can provoke strong rejection mechanisms in recipients. However, it is somewhat surprising that donor leukocytes, regardless of whether sex-mismatch or not, faired considerably worse in female recipients than in male recipients in all of the mismatched settings.

Aging also had a profound inhibitory effect on the anticancer activity of SR/CR leukocytes. The effects can be divided into two parts. The first aspect is the age of the donor leukocytes. Our data showed that old donor leukocytes had drastically reduced functionality. It is interesting to note that the age-related loss of function in the leukocytes could not be restored in the young recipients. It appeared that the functional loss in old leukocytes was persistent and structural. However, alternatively the old cells may have simply needed more time in the young environment to regain function before the challenge with the cancer cells. The second aspect is the age of recipients. Our data showed that young leukocytes were inhibited in old recipients. It seems that the young leukocytes were inhibited by either the surface molecules of the host tissues or by circulating diffusible host molecules in body fluids. It has been previously shown that the circulation of old animals contains inhibitory components that affect young tissues and cells [11]. Our observations are consistent with the theme that there are cytotoxic or inhibitory factors in the circulation of old hosts that can abolish the functionality of transplanted young donor leukocytes. One of the intriguing findings was that the deleterious effects of the host environment were not immediate. We observed a period of time during the initial 2-3 weeks, before the subsequent S180 challenges, in which the mice survived the initial S180 challenge similarly to the control. This observation suggests that during the initial 2-3 weeks, the donor leukocytes could function normally before their eventual decline.

Cryopreservation of donor leukocytes is a convenient way of retaining cell viability for a long period of time [13,14,16]. However, it was unclear whether the functionality of the stored leukocytes could be maintained. Our studies showed that the anticancer activity of donor leukocytes could be largely maintained during a period of storage and through the processes of freezing and thawing. This observation may open up the possibility of leukocyte banking from young donor leukocytes for either autologous or allogeneic use in the future [16].

Our results also showed substantial inhibition of donor cell functionality by irradiation. Although the anticancer activity of donor leukocytes does not appear to require additional cell proliferation, the effect of irradiation on donor cell function was considerable. Irradiation at high doses, such as 25 Gy, is known to induce DNA lesions in treated cells [17]. While this treatment can prevent cell proliferation, it can also shut down transcription since intact transcripts may not be able to be generated due to DNA damage. Our unpublished results show that SR/CR leukocytes have a vastly different transcription profile compared to WT leukocytes, and alteration or impairment of gene expression as a result of radiation may cause damage to transcripts required for SR/CR leukocyte killing of cancer cells.

It was encouraging to see that there was a time period during the initial 2-3 weeks in which the recipients' survival was comparable to the controls again suggesting that even in the presence of inhibitory conditions the donor leukocytes were still able to function. The donor leukocytes of the SR/CR mice displayed a meaningful anticancer functionality during this initial time period before their eventual decline. The most likely explanation for this is that the protective and therapeutic functions of SR/CR donor leukocytes against cancer can be accomplished within a few hours or days. This timeframe is well before the host's inhibitory factors have had a chance to abolish leukocyte function. Therefore, it is possible that donor leukocytes may offer a new therapeutic concept in the human setting, in which the recipients would be much older than the donors and the donor-recipient pair would most likely be unavoidably and intentionally mismatched. However, even with these suboptimal conditions, donor leukocytes may still be able to function long enough to provide a beneficial effect.

## Methods

### Cell Lines and Mouse Strains

The S180 cell line was obtained from the American Type Culture Collection (ATCC) (Manassas, VA). S180 cells were propagated in Dulbecco's Modified Eagle Medium (DMEM) with 10% fetal bovine serum (FBS) at 37°C in 5% carbon dioxide, or maintained by serial passage in wild-type (WT) C57BL/6 mice as ascites tumors. C57BL/6 (H-2b) and BALB/c (H-2d) mice were purchased from The Jackson Laboratory and Charles River, respectively. SR/CR mice in C57BL/6 (H-2b) and BALB/c (H-2d) backgrounds were bred at the animal resource program (ARP) facility of Wake Forest University School of Medicine. All mice were housed in plastic cages covered with individual air filter tops, containing corncob bedding, allowed free access to water and chow diet, and exposed to a 12-h fluorescent light/dark cycle. All procedures performed on the mice were in compliance with the guidelines approved by the Institutional Animal Care and Use Committee (IACUC) of Wake Forest University and the National Institutes of Health Guide for the Care and Use of Animals.

### Adoptive Transfer

For adoptive transfers, donor leukocytes were harvested from the spleens and bone marrow of sacrificed SR/CR mice, pooled in phosphate buffered saline and counted. The various experimental setups used one donor mouse per recipient with the exception of the mismatch experimental setup, which used one donor per recipient for the male donors and approximately 1.5 donors per recipient for the female donors due to differences in the spleen sizes. The number of leukocytes transferred to each donor was well above the minimum number of transferred leukocytes (5 × 10^6^) that are necessary to transfer the SR/CR phenotype (unpublished data). The number of transferred cells was matched within each experimental group based on a cell count or approximate spleen size and ranged from 51 × 10^6 ^to 102 × 10^6 ^or 1 to 1.5 spleens per recipient. The exception to this range and matching criteria were the cryopreservation adoptive transfer experimental group which had an approximately 2 × 10^6 ^difference between the control and treated group (7-9 × 10^6 ^for each recipient). The pooled donor leukocytes were then treated, if applicable, and injected intraperitoneally (IP) into recipient mice. One day after the leukocyte transfers, recipient mice were challenged with 1 × 10^6 ^S180 to verify resistance. As previously shown [2], recipient mice survived the S180 challenges only if the transfers of the SR/CR leukocytes were successful.

### Irradiation

When desired, leukocytes from donor mice were irradiated in flasks with a total accumulated dose of 25 Gray (Gy) with a cesium irradiator.

### Cryopreservation

For cryopreserving, donor leukocytes were resuspended in a cryopreserving media consisting of 90% FBS +10% Dimethyl Sulfoxide (DMSO) and stored first in a -80°C freezer for approximately one day before being transferred to liquid nitrogen for approximately one month. Before transfers, the stored leukocytes were thawed in a 37°C water bath, washed two times with phosphate buffered saline to remove any remaining fetal bovine serum, and counted via trypan blue exclusion.

### Histological Analysis

At desired time points of the studies, mice were sacrificed. The lungs, livers, intestines, kidneys, spleens and other tissues of potential interest were dissected and stored in 10% neutral buffered formalin. The collected tissues were embedded in paraffin. Sections of the tissues were stained with hematoxylin and eosin and examined for histological abnormalities.

## Competing interests

The authors declare that they have no competing interests.

## Authors' contributions

JS helped conceive of the study, participated in its design and coordination, helped perform the adoptive transfers, irradiation, and histological analysis and drafted the manuscript. MB helped design the studies, perform the adoptive transfers, and with manuscript and figure preparation. GR helped design the studies, perform the adoptive transfers, and helped with manuscript preparation. JK performed the histological evaluation of the stored tissues and helped with manuscript preparation. AS helped design the studies, perform the adoptive transfers, and with manuscript preparation. JA helped perform the adoptive transfers and animal care and helped with manuscript preparation. MW helped design the studies, helped with the histological evaluation, and with manuscript preparation. ZC helped conceive and design the studies and with drafting the manuscript. All authors read and approved the final manuscript.

## Pre-publication history

The pre-publication history for this paper can be accessed here:

http://www.biomedcentral.com/1471-2407/9/328/prepub
